# Floral Induction and Flower Development of Orchids

**DOI:** 10.3389/fpls.2019.01258

**Published:** 2019-10-10

**Authors:** Shan-Li Wang, Kotapati Kasi Viswanath, Chii-Gong Tong, Hye Ryun An, Seonghoe Jang, Fure-Chyi Chen

**Affiliations:** ^1^Biotechnology Center in Southern Taiwan (BCST) of the Agricultural Biotechnology Research Center (ABRC), Academia Sinica, Tainan, Taiwan; ^2^Department of Plant Industry, National Pingtung University of Science and Technology, Pingtung, Taiwan; ^3^National Institute of Horticultural and Herbal Science (NIHHS), Rural Development Administration (RDA), Wanju-gun, South Korea; ^4^World Vegetable Center Korea Office (WKO), Wanju-gun, South Korea

**Keywords:** floral transition, flower development, molecular genetics, orchids, orchid biotechnology, transgenic orchids

## Abstract

Orchids comprise one of the largest, most highly evolved angiosperm families, and form an extremely peculiar group of plants. Various orchids are available through traditional breeding and micro-propagation since they are valuable as potted plants and/or cut flowers in horticultural markets. The flowering of orchids is generally influenced by environmental signals such as temperature and endogenous developmental programs controlled by genetic factors as is usual in many flowering plant species. The process of floral transition is connected to the flower developmental programs that include floral meristem maintenance and floral organ specification. Thanks to advances in molecular and genetic technologies, the understanding of the molecular mechanisms underlying orchid floral transition and flower developmental processes have been widened, especially in several commercially important orchids such as *Phalaenopsis*, *Dendrobium* and *Oncidium*. In this review, we consolidate recent progress in research on the floral transition and flower development of orchids emphasizing representative genes and genetic networks, and also introduce a few successful cases of manipulation of orchid flowering/flower development through the application of molecular breeding or biotechnology tools.

## Introduction

The orchid family is one of the largest families of angiosperms. The flowers of Orchidaceae exhibit a high degree of speciation, with wide variations in floral features such as morphology, color, size and fragrance for attraction of pollinators (Peakall, 2007). Orchids are traded worldwide as cut flowers and potted flowering plants. The flowering of orchids can generally be divided into two steps: floral transition and flower development. In floral transition, juvenility, ambient to cool temperature and/or photoperiod are crucial in determining the time that orchids initiate flowering with respect to ontogeny and season. Although molecular and genetic approaches have made it possible to shed some light on the mechanisms underlying floral transition in model plants such as *Arabidopsis* (*Arabidopsis thaliana*) and rice (*Oryza sativa*) (He, 2009; Sun et al., 2014), orchid flowering is still not well-understood. Flower formation is initiated by activities of genes known as flowering time genes which regulate the conversion of the vegetative meristem to the floral meristem. Then, floral meristem identity genes regulate the formation of the flower. Once flowering initiates, cadastral genes which govern the whorl formation in flowers are expressed, and the structures of the whorls and their occurrence at the right position are regulated by homeotic genes. Although extreme variation in flower morphology is found across the angiosperms, four types of relatively simple floral organs can be distinguished, namely sepals, petals, stamens and carpels (Theissen and Melzer, 2007; Heijmans et al., 2012). Based mainly on studies with *Arabidopsis*, the ABCDE model explains the establishment and maintenance of floral organ specification through an interactive network of MADS-box transcription factors. Herein, we review recent research on floral transition and flower development of a few representative orchid species from the perspective of molecular genetics. Some successful cases of the use of biotechnological tools to modulate orchid floral traits are also highlighted.

## Floral Transition of Orchids

### Effect of Ambient Temperature

The Orchidaceae plants are widely distributed across the globe, thus the mechanisms of flowering control among different genera may have developed according to their natural habitats. Apostasioideae, Cypripedioideae, Vanilloideae, Epidendroideae and Orchidoideae are the subfamilies of Orchidaceae. Among these five subfamilies, the Epidendroideae is the largest containing more than 500 genera with around 20,000 species (Freudenstein and Chase, 2015). Different orchids of the Epidendroideae require different conditions of ambient temperature and photoperiod to induce flowering (Hsiao et al., 2011).

Most orchids take several years to finish the juvenile stage. *Phalaenopsis* usually reach maturity after three to five leaves. Usually, the floral spike (inflorescence) emerges from the axillary buds of the fourth node below the apical leaf whereas other axillary buds are maintained in a dormant state during the flowering season (Sakanishi et al., 1980). The mechanism of differential commitment of distinct axillary buds for flowering is unknown. At the beginning of flowering, the axillary buds are enlarged and then protrude from the base of a leaf, which takes about 3–4 weeks. After that, the bud is elongated to become a floral spike.

The flowering of several orchids is influenced by change in ambient temperature. The flowering of *Phalaenopsis* is promoted by low ambient temperature, usually less than 26 °C (Blanchard and Runkle, 2006); and can be reversed if the ambient temperature is elevated. The flowering activity of *Dendrobium* is also promoted by low ambient temperature (Campos and Kerbauy, 2004); however, flowering of some hybrid cultivars of *Dendrobium*– such as *Dendrobium* Chao Praya Smile and *Dendrobium* Madame Thong-In, are promoted by high ambient temperature. The flowering of *Miltoniopsis* and *Zygopetalum* are promoted by cool temperature 11–14 °C (Lopez et al., 2003; Lopez and Runkle, 2006). For the *Oncidium*, another popular orchid, change in ambient temperature enhances or is neutral to their flowering activity. For *Cypripedium* species, vernalization below 5 °C or subzero temperature is required for flowering (Zhang et al., 2014).

In the model plant *Arabidopsis*, flowering activity is delayed by low ambient temperature (16 °C) but promoted by vernalization. In low ambient temperature, an alternatively spliced form of FLOWERING LOCUS M (FLM), FLM-β associates with SUPPRESSOR OF VEGETATIVE PHASE (SVP) to suppress the floral integrator gene *FLOWERING LOCUS T* (*FT*) (Lee et al., 2013). The expression of *FLM* is down-regulated *via* alternative splicing coupled with nonsense-mediated decay (AS-NMD) in response to elevated temperatures (Sureshkumar et al., 2016), indicating that *FLM* plays an important role in flowering initiation by modulating thermo-sensitivity. The *FT* expression is also suppressed by a MADS-box gene *FLOWERING LOCUS C* (*FLC*). Constant expression of *FLC* prevents the winter-annual *Arabidopsis* from floral transition before winter and this repression is released by the vernalization pathway signaling (He, 2009). The vernalization pathway suppresses the *FLC* activity *via* the trimethylation of histone 3 lysine 27 (H3K9me3) of *FLC* chromatin. Vernalization is an obligatory requirement for the flowering of lily (*Lilium longiflorum*). In high ambient temperature, a fully developed floral bud is present inside the bulb. A period of prolonged cold temperature in winter is required for the switch of shoot apical meristem (SAM) to inflorescence meristem (IM). The candidate genes involved in vernalization response in lily have been elucidated using transcriptome analysis (Villacorta-Martin et al., 2015; Li et al., 2016) and several orthologs of *Arabidopsis* flowering genes were identified. Among them, *SVP* and *VERNALIZATION 1* (*VRN1*) were down- and up-regulated respectively in floral buds when the bulbs were treated with low ambient temperature. Furthermore, ectopic expression of lily *SVP* or *VRN1* in *Arabidopsis* resulted in delayed or earlier flowering, respectively.

Understanding how ambient temperature regulates the floral transition of orchids is valuable not only for the horticultural market but also for the comprehension of flowering regulation in different plant species. Using molecular genomics tools to investigate flowering in popular ornamental orchids has made it possible to identify many flowering-related genes; however, how such genes regulate flowering in an ambient temperature-dependent manner is still not clear.

### Effect of Photoperiod

In *Arabidopsis* and rice, photoperiod is crucial for flowering control (He, 2009; Sun et al., 2014; Song, 2016). Under long-day conditions, *Arabidopsis FT* expression is activated in vascular tissues of leaves in a circadian rhythmic manner by the transcriptional regulator CONSTANS (CO) whose expression and activity are controlled by light signaling pathways and the circadian clock (Song, 2016). In rice, two orthologs of *FT*, *Heading date 3a* (*Hd3a*) and *RICE FLOWERING LOCUS T* (*RFT*) are mainly responsible for floral induction under short-days (SD) and LD conditions, respectively (Sun et al., 2014).

Floral initiation has been shown to be regulated by photoperiod in a few orchids. In *Doritis pulcherrima* (now *Phalaenopsis pulcherrima*), under the 30 °C/20 °C (day/night) condition, floral spikes are initiated more efficiently by 9-h light than 12-h light treatment (Wang et al., 2003). In *Miltoniopsis* orchids, SD incubation at 23 °C before shifting to cool temperature (11–14 °C) facilitates flowering (Lopez and Runkle, 2006). However, during cool temperature treatment, different photoperiods have no significant effect on flowering suggesting that ambient temperature may play a major role in flowering of *Miltoniopsis*. On the other hand, the formation of floral spikes in *Psygmorchis pusilla* is positively correlated with the increase in day length implying that *P. pusilla* is a quantitative LD plant (Vaz et al., 2004). The effect of photoperiod on flowering seems to be various among different orchid species which have great diversity in adaptation. Furthermore, most orchids are native to tropical areas where day length does not change dramatically throughout the year. Thus, it is reasonable to anticipate that photoperiod has limited effects on flowering of orchids.

### Effect of Phytohormones

The effects of phytohormones on orchid flowering have been studied (Goh and Yang, 1978). A synthetic cytokinin, 6-benzylaminopurine (BA), stimulated the flowering of monopodial (e.g., *Phalaenopsis*) and sympodial (e.g., *Dendrobium*) orchids whereas auxin suppressed the BA effect. The positive effect of BA on flowering is likely to be enhanced when combined with gibberellic acid (GA_3_) although GA_3_ applied alone does not have an influence on floral induction (Hew and Clifford, 1993). In *Phalaenopsis* and *Doritaenopsis*, plants sprayed with BA produced visible inflorescences 3 to 9 days earlier than those without BA treatment although the application of BA could not replace the inductive low temperature in *Phalaenopsis* (Blanchard and Runkle, 2008). Interestingly, even though GAs do not induce flowering, optimum levels of endogenous GAs in the flowering shoot tips are required for flower development in *Phalaenopsis*. Indeed, injection of GAs can rescue the blockage of flower development under high temperature (Su et al., 2001). The existence of abscisic acid (ABA) in different tissues of *Phalaenopsis* has been examined. Relatively higher level of free ABA has been detected in dormant axillary buds whereas free or bound forms of ABA were not found in floral shoots (Wang et al., 2002). In addition, exogenous application of ABA to the stem of *Phalaenopsis* inhibited the formation of floral spikes even under inductive low ambient temperature conditions implying that ABA plays an inhibitory role in orchid floral transition.

Taken together, developmental maturity and ambient temperature are likely the main endogenous and environmental factors, respectively, controlling orchid flowering together with the combined action of phytohormones. Many orchids are epiphytic plants, meaning they are adapted to face frequent periods of nutrient and water scarcity. Although this kind of orchid uses the crassulacean acid metabolism (CAM) as the strategy for carbon fixation like other CAM plants such as succulents and pineapples to adapt themselves to arid conditions (Silvera et al., 2009), flowering consumes a lot of energy. Therefore, the right timing of bud commitment for floral induction with the best physiological situation is crucial for the maintenance of the species through successful sexual propagation. In addition, most orchids have particular pollinators in the wild environment; the timing of floral transition should be consistent with the appearance of their pollinators. Thus, sensing ambient temperature seems to be a good strategy to achieve this aim if their pollinators only appear in a particular season.

### Genes Involved in Flowering in *Phalaenopsis*

Based on expression analyses and functional studies with heterologous expression systems, candidate genes for flowering regulatory networks in orchids have been reported. Recently, genome-wide analyses have also been taken into consideration for the identification of Orchidaceae-specific and/or species-specific key genes controlling flowering/flower development of orchids (Huang et al., 2016; Lin et al., 2016; Wen et al., 2017).

*P. aphrodite FT1* (*PaFT1*) has been isolated and functionally characterized (Jang et al., 2015). Flowering of *P. aphrodite* is induced by low ambient temperature (≤25 °C) but prohibited by high ambient temperature (≥28 °C). Moreover, photoperiod has no significant influence on flowering of *P. aphrodite*. Expression patterns of *PaFT1* reflect the flowering behavior of the orchid; its flowering is induced by low ambient temperature but not by different light regimes. Reduced expression level of *PaFT1* by virus-induced gene silencing (VIGS) methods resulted in delayed flowering under inductive low ambient temperature, while ectopic expression of *PaFT1* suppressed the late flowering phenotype caused by the induced expression of *SVP* and active *FRIGIDA* (*FRI*), a *FLC* activator, in *Arabidopsis*. Moreover, PaFT1 is able to physically interact with PaFD reminiscent of the *Arabidopsis* FT-FD module (He, 2009). *LEAFY* (*LFY*) is another floral integrator in *Arabidopsis* (He, 2009). The *LFY* gene of *P. aphrodite* (*PhapLFY*) has also been characterized (Jang, 2015). Expression of *PhapLFY* under the control of *Arabidopsis LFY* promoter rescued the abnormal floral structure of *Arabidopsis lfy* mutant and caused early heading by overexpression in rice. Recently, a *CO*-like gene, *PhalCOL* has been identified in *P. hybrida* (Wedding Promenade) (Zhang et al., 2011). *PhalCOL* has significant sequence similarity to *CO* of *Arabidopsis*. Overexpression of *PhalCOL* in tobacco caused an early-flowering phenotype suggesting the functional convergence of *CO* genes in flowering among different plant species. To discover candidate genes responsible for the flowering control of *P. aphrodite*, transcription profiles in axillary buds of plants treated with low and high ambient temperature were analyzed and compared to each other (Huang et al., 2016). The result showed that, in addition to *FT*, *LFY*, *APETALA1* (*AP1*) and *OVEREXPRESSION OF CONSTANS 1* (*SOC1*) homologs, genes involved in GA biosynthesis were also up-regulated by low ambient temperature. Another expression analysis using *Phalaenopsis* Fortune Saltzman found that transcripts of *KNOX1*, R2R3-like *MYB*, adenosine kinase 2, S-adenosylmethionine synthetase, dihydroflavonol 4-reductase, and naringenin 3-dioxygenase were accumulated at a higher level in spikes grown under warm day/cool night (28 °C/21 °C) compared with those grown at daily warm temperature (28 °C/26 °C) under natural light conditions (Li et al., 2014).

### Genes Involved in Flowering in *Dendrobium*

Flowering of *D. nobile* is promoted by low ambient temperature (Campos and Kerbauy, 2004). Orthologs of *FT* and *MOTHER OF FT AND TFL1* (*MFT*), a homolog of *FT*, have been identified in *D. nobile* Lindl. (Li et al., 2012). Expression of *DnFT* is up-regulated in leaves but down-regulated in axillary buds under low temperature treatment (12 °C/9 °C, day/night) whereas *DnMFT* expression is not affected by the low temperature treatment. Transgenic *Arabidopsis* ectopically expressing *DnFT* exhibited early flowering. In addition, an ortholog of *SOC1*, *DOSOC1* has been identified from *Dendrobium* Chao Praya Smile (Ding et al., 2013). Increased expression of *DOSOC1* was detected during floral transition. Moreover, overexpression of *DOSOC1* in both *Arabidopsis* and *Dendrobium* resulted in early-flowering phenotypes. *DOAP1*, an ortholog of *AP1* has also been identified and characterized from the orchid (Sawettalake et al., 2017). The role of *DOAP1* is similar to that of *AP1* in *Arabidopsis*. Overexpression of *DOAP1* rescued the floral defect of *ap1* mutant and resulted in early flowering in wild-type *Arabidopsis*. Differential gene expression in the SAM during *in vitro* transition from vegetative to reproductive growth has been investigated in *Dendrobium* Madame Thong-In (Yu and Goh, 2000a). Several transcription factors, including a MADS-box gene of the *AP1*/*AGL2* family, a class I *KNOX* gene and a homolog of the *Drosophila* *SEVEN-UP* gene were differentially expressed in vegetative and transitional SAM. The *KNOX* gene plays an important role in the function of SAM, and encodes a KNOTTED1-like homeobox (Knox) protein later designated as *DOH1* (*Dendrobium Orchid Homeobox 1*) (Yu et al., 2000). In tissue culture conditions, the expression of *DOH1* was gradually increased in the apical meristem during vegetative growth whereas it was decreased during the progress of reproductive growth. Overexpression of antisense *DOH1* resulted in early flowering in *Dendrobium*. On the other hand, *DOMADS1*, a MADS-box gene of the *AP1*/*AGL9* superfamily, was identified from the cDNA library of transitional SAM (Yu and Goh, 2000b). Expression of *DOMADS1* was up-regulated in transgenic plants harboring p*35S*: antisense *DOH1* suggesting that *DOH1* is a possible upstream repressor of *DOMADS1* in the flowering control of *Dendrobium* orchids. Recently, transcriptomes of *D. nobile* were analyzed under cold (4 °C) or exogenous cytokinin (thidiazuron) treatment (Wen et al., 2017). The results showed that *SOC1*, *LFY* and *AP1* genes were induced by both low temperature and thidiazuron treatment whereas *DnVRN1* and *FT* were induced only by cold treatment. Also, some marker genes for the GA signaling pathway were up-regulated under both low temperature and thidiazuron treatments. Further investigation is needed to uncover the cytokinin-GA signaling network in the inductive temperature condition underlying the floral transition in *Dendrobium*.

### Genes Involved in Flowering in *Oncidium*

High ambient temperature (30 °C) accelerates the flowering of *Oncidium*. The flowering response to changes in ambient temperature in *Oncidium* is opposite to that of *Phalaenopsis*. The flowering promoter *FT* and repressor *TERMINAL FLOWER 1* (*TFL1*) both encode proteins belonging to phosphatidylethanolamine-binding protein (PEBP) family and have also been identified in *Oncidium* Gower Ramsey (Hou and Yang, 2009). *OnFT* was expressed in axillary buds, leaves, pseudo-bulb and flowers while *OnTFL1* was only expressed in axillary buds and pseudo-bulb. The expression of *OnFT* was regulated by photoperiod, with highest expression from the 8th to 12th hour of the light period and lowest expression at dawn. However, expression of *OnTFL1* is not influenced by photoperiod. Ectopic expression of *OnFT* resulted in an early-flowering phenotype in *Arabidopsis*, and late-flowering of *Arabidopsis ft-1* mutant was rescued by *OnFT* overexpression (Hou and Yang, 2009). On the contrary, ectopic expression of *OnTFL1* in *Arabidopsis* displayed late flowering. *OMADS1*, a homolog of *Arabidopsis* *AGL6* has also been identified in *Oncidium* Gower Ramsey (Hsu et al., 2003). *OMADS1* transcripts were detectable in the apical meristem and floral organs, and ectopic expression of *OMADS1* in *Arabidopsis* caused early flowering with up-regulation of *FT*, *SOC1*, *LFY* and *AP1*. Overexpression of *OMADS1* in *Oncidium* also resulted in precocious flowering (Hsu et al., 2003). Recently, ascorbate (AsA) content has been shown to play an important role in floral transition in response to thermal stress (30 °C over a 14-day period) in *Oncidium* Gower Ramsey (Chin et al., 2014). Under thermal stress conditions, the level of reactive oxygen species (ROS; e.g., H_2_O_2_) was significantly increased and the AsA redox ratio [AsA to dehydroascorbate (DHA, the oxidized form of AsA)] was decreased with prominent up-regulation of cytosolic ascorbate peroxidase (*cytAPX1*) expression. The oxidation of AsA to DHA by ascorbate peroxidase is the key reaction to remove H_2_O_2_. The report by Chin and colleagues suggests that the AsA/DHA redox ratio may act as one of the endogenous signals that induce the flowering of *Oncidium* in response to high ambient temperature. Furthermore, it has been shown that reduced GSH redox ratio caused by down-regulation of GSH metabolism-related genes such as glutathione reductase (*GR1*), γ-glutamylcysteine synthase (*GSH1*) and glutathione synthase (*GSH2*) was linked to the decrease in the AsA redox ratio for flowering of *Oncidium* orchid (Chin et al., 2016).

### Genes Involved in Flowering in *Dortiaenopsis* and Other Orchids

*DhFVE*, a *Dortiaenopsis* ortholog of *Arabidopsis FVE*, which is a component of the autonomous flowering pathway, has been identified (Sun et al., 2012). Flowering of *Dortiaenopsis* (now *Phalaenopsis*) is promoted by low ambient temperature (22 °C/18 °C, day/night) (Luo et al., 2011) and the expression of *DhFVE* is increased in the stem during floral transition. In addition, *EARLY FLOWERING 4* (*EFL4*) family genes, *DhEFL2*, *DhEFL3* and *DhEFL4* have been identified in *Dortiaenopsis* (Chen et al., 2015). *Arabidopsis EFL4* is known to affect flowering through photoperiod perception and circadian regulation (Doyle et al., 2002). Ectopic expression of *DhEFL2*, *DhEFL3* or *DhEFL4* delayed flowering of *Arabidopsis*. Moreover, *GIGANTEA* (*GI*), an upstream activator of *CO* (Sawa et al., 2007), has been identified as *DhGI1* in *Dortiaenopsis* hybrid (Luo et al., 2011). Expression of *DhGI1* is up-regulated by low temperature and may play a role in flowering initiation in *Dortiaenopsis* hybrid. Recently, many putative flowering genes have been identified in *Cymbidium* and *Erycina* through transcriptome analyses. However, their functions still remain to be examined (Li et al., 2013; Lin et al., 2016).

### A Hypothetical Model of Flowering Regulation in *P. aphrodite*

The regulatory networks of floral transition in different orchids may be divergent since the required inductive conditions are not all the same. In addition, the genetic tools for orchid research are still limited; therefore, it is time-consuming and also difficult to reveal the molecular mechanisms of flowering control in orchids. The popular ornamental orchid *P. aphrodite* has a particular requirement for floral transition, i.e., low ambient temperature, which makes this orchid an interesting target for a case-study of flowering regulation in orchids. Furthermore, a transformation system has been established in orchids including *P. aphrodite* (Hsing et al., 2016). Thus, the molecular mechanism of flowering regulation could be investigated more thoroughly.

**Figure 1:**
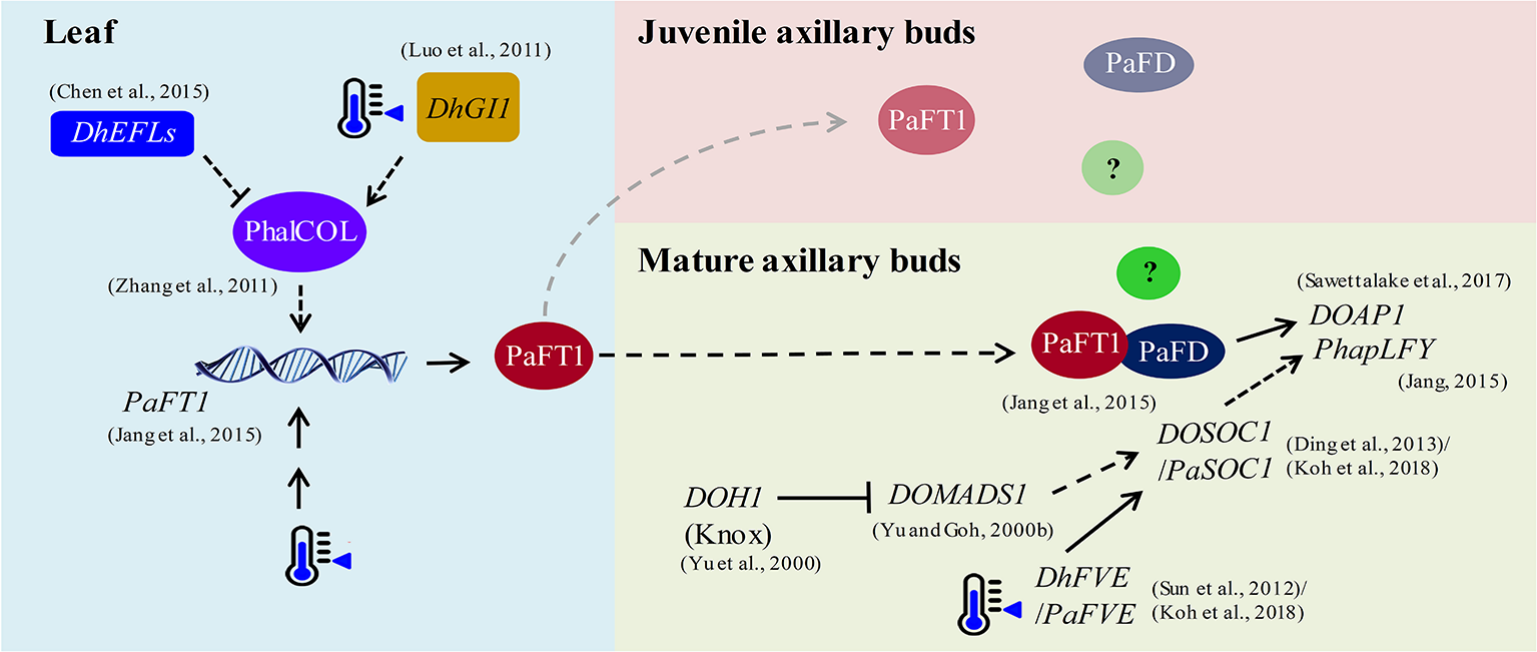
The hypothetical flowering regulation in *P. aphrodite* under low ambient temperature. Low ambient temperature drives *PaFT1* activity in leaves and then PaFT1 protein is transported selectively to mature axillary buds or both mature and juvenile axillary buds. A complex formed by the interaction between PaFT1 and PaFD may activate downstream genes such as *AP1* required for floral meristem identity. An example of unknown factors for flowering in the meristem is presented by a green circle with a question mark inside. In juvenile axillary buds, some flowering activators required for floral induction may be absent. *PaFT1* can also be activated by *CO* pathway and the *CO* activity—*PhalCOL* (*Phalaenopsis CO Like*), here—which is likely to be regulated by *EFL* and *GI* genes. This regulation should not be dependent on photoperiod. Low ambient temperature also activates *PaFVE*. Consequently, *PaSOC1* is activated by the *PaFVE*. Moreover, the reduced expression of a KNOTTED1-like homeobox (Knox) gene may lead to the activation of *SOC1* through other MADS box genes such as *DOMADS1*. The orchid *LFY* genes such as *PhapLFY* can also be induced by *PaSOC1*. The solid lines represent pathways that have been reported in orchids and the dotted lines indicate hypothetical regulations based on studies using other plant species. References for each regulation are presented in the figure.

The hypothetical gene regulatory network in flowering of *P. aphrodite* is illustrated in **Figure 1** based on the published results (data adapted from research on orchids). The expression of *PaFT1* is induced by low ambient temperature, and PaFT1 interacts with PaFD to possibly activate the downstream genes required for floral induction. Reflecting the *Arabidopsis* model, PaFT1 protein is likely transported from leaves to axillary buds to induce spiking in *Phalaenopsis* orchid. If the PaFT1 moves to dormant/juvenile buds, there must be a repressive mechanism against PaFT1 action or other unknown floral co-activators are still absent in dormant/juvenile buds. However, we cannot exclude the possibility that the pavement for the PaFT1 movement to the dormant/juvenile buds is not available. Based on the transcriptomic analyses of *Phalaenopsis* orchids, *KNOX1*, *SOC1* and *FVE* genes are induced by low ambient temperature (Sun et al., 2012; Li et al., 2014; Huang et al., 2016) although *Dendrobium KNOX1* gene is down-regulated by low ambient temperature (Yu and Goh, 2000a; Yu et al., 2000). Thus, the role of *KNOX1* in floral transition of *Phalaenopsis* needs to be further examined. In *Phalaenopsis*, *SOC1* may activate *LFY* and *AP1* during floral transition as is the case in *Arabidopsis*. Recently, *FVE* has been suggested to be an upstream activator of *SOC1* in *P. aphrodite* (Koh et al., 2018). The *CO* in *Phalaenopsis* may also activate *FT* irrespective of photoperiod (Zhang et al., 2011; Kaewphalug et al., 2017). In addition, *ELF* genes in *Phalaenopsis* may repress the *CO* activity (Doyle et al., 2002; Chen et al., 2015). Moreover, the GI-FLAVIN-BINDING, KELCH REPEAT, F BOX 1 (FKF1) and CYCLING DOF FACTOR (CDF) may also regulate the *CO* activity in *Phalaenopsis* as is also the case in *Arabidopsis* (Song, 2016). Since *SVP* and *FLM* are critical flowering regulators responding to changes in ambient temperature in *Arabidopsis*, it is also possible to anticipate that the two orthologs of *SVP* and *FLM* also act in floral induction of *Phalaenopsis* in various ways. Actually, *SVP* and *FLM* repress the expression of *FT* at low ambient temperature in *Arabidopsis* (Lee et al., 2013). Thus, the working mechanism of those orthologs in *Phalaenopsis* might be distinct from that of *Arabidopsis*. In addition, further studies are required to reveal the mechanisms underlying phytohormone-dependent flowering pathways linked to changes in ambient temperature in orchids. Even taking these findings together, it can be seen that there is a long way to go to achieve a better understanding of the flowering regulatory network in *P. aphrodite*. As one of the shortcuts to reach this goal, high throughput analyses can be applied to identify candidates involved in the floral transition of *Phalaenopsis*. Also, collection of information on flowering networks in various plant species would be very useful in the interpretation of large-scale experimental results such as omics data. Most importantly, forward or reverse genetic studies are required for the functional confirmation of candidate genes in orchid flowering. The homologs of flowering genes in a few orchid species are listed in **Table S1**.

## Flower Development of Orchids

### Floral Development of *Arabidopsis* and Monocot Plants

**Figure 2:**
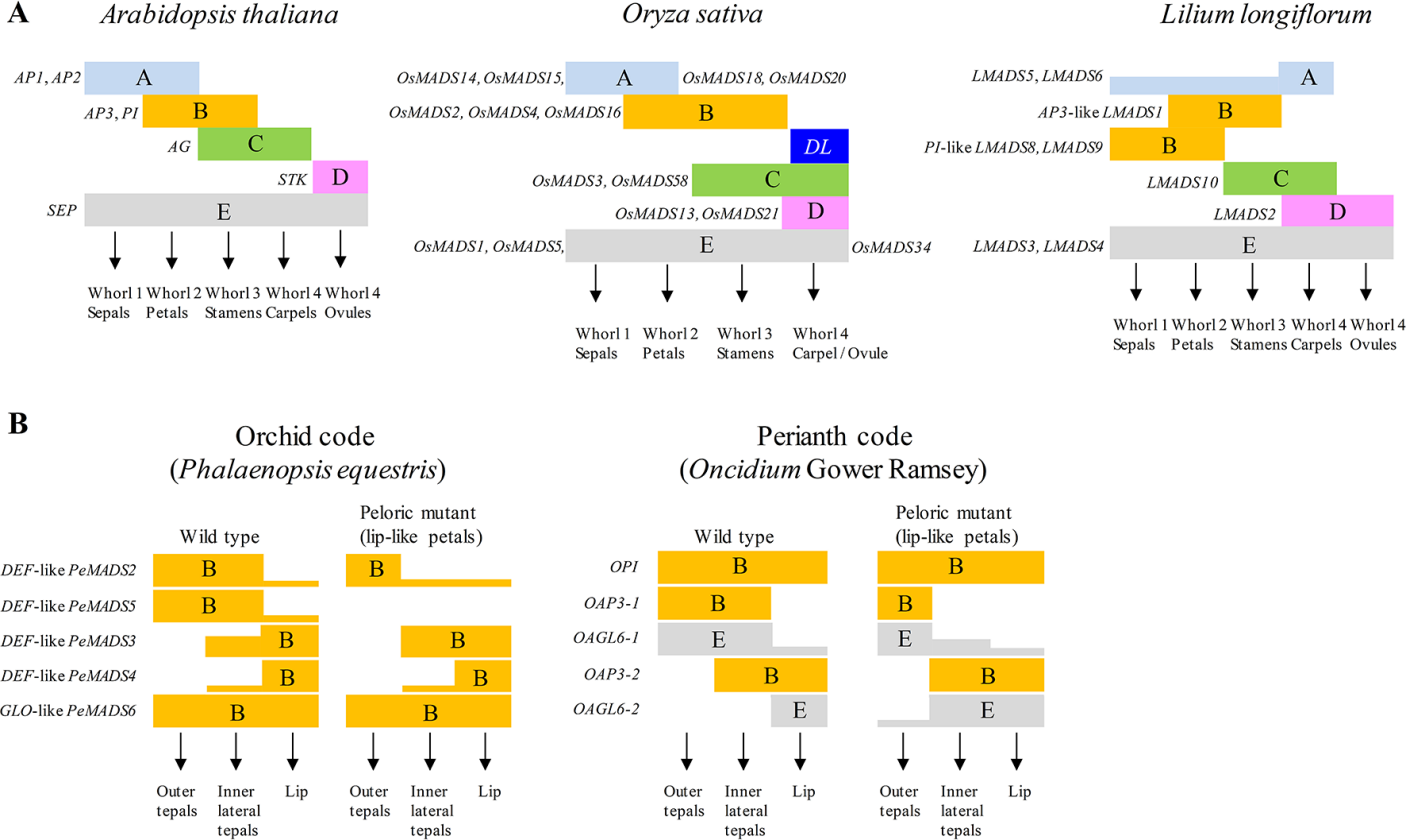
The models of floral development in *Arabidopsis*, rice, lily and orchids. **(A)** The ABCDE model explains the floral organ identity in *Arabidopsis*, rice and lily. The floral development genes are grouped into different classes from A to E. Combinational expressions of different classes of genes determine the formation of different floral organs. Representative genes of different classes are indicated in the figures. *DL*, *DROOPING LEAF*. **(B)** The orchid code and perianth code (P code) explain the formation of perianth of orchids. In the orchid code, combinational expressions of B-class genes from different phylogenetic clades determine the morphology of orchid perianth. The expression patterns of B-class genes changed in peloric mutant are compared with that of wild type. In the P code model, combinational expressions of different B and E-class genes determine the formation sepal/petal or lip of *Oncidium* orchids.

Floral pattern formation is one of the significant features of angiosperms, the mechanisms of which can be explained using the ABC or ABCDE model (**Figure 2A**). The identity of various floral organs is determined by MADS-box transcription factors or their complexes during flower development. According to the ABC model, in *Arabidopsis*, sepals in the first whorl are characterized by the expression of A-class genes; petals in whorl 2 are characterized by co-expression of A- and B-class genes, stamens in whorl 3 are characterized by co-expression of B- and C-class genes, and carpels in whorl 4 are determined when C-class genes are solely expressed (Jack, 2001; Theissen, 2001). The ABC model has been extended to the ABCDE model with additional functions of D- and E-class genes in floral organ identity determination (Theissen and Melzer, 2007; Heijmans et al., 2012). D-class genes are involved in ovule development while E-class genes are involved in the identity of all floral verticils. Most genes in the ABCDE model are MADS-box genes. *AP1* and *AP2* (the only non-MADS-box genes) are A-class genes; *AP3* and *PISTILLATA* (*PI*) are B-class genes. *AGAMOUS* (*AG*) is a C-class gene; *SEEDSTICK* (*STK*) is a D-class gene. The D-class genes were first identified in *Petunia*, namely *FLORAL BINDING PROTEIN 7* (*FBP7*) and *FBP11*. Finally, *SEPALLATA 1* (*SEP1*), *SEP2, SEP3* and *SEP4* formally known as *AGAMOUS-LIKE 2* (*AGL2*), *AGL4*, *AGL9* and *AGL3*, respectively, are characterized as E-class genes. According to the floral quartet model, MADS-box proteins form whorl-specific tetrameric complexes during floral organ determination. The tetrameric transcriptional factor complexes recognize specific *cis*-regulatory elements termed CArG-boxes (CC-AT rich-GG), and different complexes are postulated to function in controlling expression of different target genes (Jetha et al., 2014). Recently, a reliable and efﬁcient two-stage approach has been developed for angiosperms MADS-Box genes classification using machine learning methods to overcome errors in phylogenetic tree construction (Chen et al., 2019).

The MADS-box genes involved in the floral development in monocot plants such as rice, maize (*Zea mays*), wheat (*Triticum aestivum*), barley (*Hordeum vulgare*) and lily have also been elucidated (Callens et al., 2018). MADS-box protein structure is conserved between diverse plants. Comparing the expressions and functions of the ABCDE MADS-box genes among different monocot plants provides the opportunity to elucidate their roles in determining the floral development during evolution. In rice, the A-class gene, the *FUL*-like (*FRUITFULL* locus of *Arabidopsis*) *OsMADS14*, *OsMADS15*, *OsMADS18* and *OsMADS20*, are expressed to determine the whorl 1 structures of lemma and palea; co-expression of A and B-class genes, *DEF*-like (*DEFICIENS*: an *Antirrhinum* B-class gene) *SUPERWOMEN1* (*SPW1 or OsMADS16*) and *GLO*-like (*GLOBOSA*: another *Antirrhinum* B-class gene) *OsMADS2* and *OsMADS4*, determines the whorl 2 structure of lodicules (Yoshida and Nagato, 2011); co-expression of B and C-class genes, *OsMADS3* and *OsMADS58*, determines the whorl 3 structure of stamens. Another C function gene *DROOPING LEAF* (*DL*) have been identified in carpel specification and floral meristem determinacy (Shige-Hiro et al., 2019). C and D-class genes, *OsMADS13* and *OsMADS21*, are expressed to determine the whorl 4 structure of the carpel and ovule, respectively. In addition, the E-class genes, *OsMADS1*, *OsMADS5* and *OsMADS34*, are involved in the development of all four whorl structures (**Figure 2A**).

The lily has a floral character most similar to orchids, with almost identical sepals and petals. Flowers from the lily family *Liliaceae* have three sepals in whorl 1, three petals in whorl 2, six stamens in whorl 3 and three fused carpels in whorl 4. Three A-class genes from lily, *LMADS5*, *LMADS6* and *LMADS7* have been analyzed (**Figure 2A**) (Chen et al., 2008). They were all expressed in the vegetative stem and inflorescence meristem. With regard to the floral organs, *LMADS5* and *LMADS6* are mainly expressed in the carpel, whereas *LMADS7* is only expressed in the vegetative stem and inflorescence meristem. Early flowering phenotype and homeotic conversions of sepals to carpel-like and of petals to stamen-like structure were observed when they were ectopically expressed in *Arabidopsis*. For the B-class gene, mRNA of *AP3*-like *LMADS1* could be detected in all four whorls but the protein was detected only in the petal and stamen (Tzeng and Yang, 2001). The *PI*-like *LMADS8* and *LMADS9* are expressed in whorl 1 and 2 during all stages of development and are expressed in the stamen only in young flower buds (Chen et al., 2012a). The ectopic expression of *LMADS8* and *LMADS9* partially converted the sepals into petaloid structures in *Arabidopsis*. The *PI*-like gene *LFGLOA* and *LFGLOB* identified from *L*. x *formolongi* are also expressed in the first, second and third whorl of lily (Akita et al., 2008). Several C-class genes have been identified from different lilies including *LMADS10*, *LFAG1* and *LLAG1* (Benedito et al., 2004; Akita et al., 2008; Hsu et al., 2010). They are expressed in whorl 3 and 4, and ectopic expression of C-class genes in *Arabidopsis* resulted in carpel-like septal and stamen-like petal phenotypes. In addition, a double-flowered cultivar “Aphrodite” of *L*. x *formolongi* with petal-like stamen may be caused by reduction in the expression of *LFAG1* in whorl 3 (Akita et al., 2008). The lily D-class gene, *LMADS2* completely accumulated in the carpel and more specifically in the ovules (Tzeng et al., 2002). Ectopic expression of *LMADS2* in *Arabidopsis* converted the sepals into a stamen-like structure in whorl 2. *LMADS3* and *LMADS4* have been identified as E-class genes in lily (Tzeng et al., 2003). Both *LMADS3* and *LMADS4* expression are expressed in the inflorescence meristem, floral buds and all four whorls of the flower organ. Ectopic expression of *LMADS3* in *Arabidopsis* showed extremely early flowering, which was associated with up-regulation of *FT*, *SOC1*, *LUMINIDEPENDENS* and flower meristem identity gene *LFY* and *AP1*. Taken together, the different expression patterns of A- and B-class genes from that of the dicot plants such as *Arabidopsis* suggest a modified ABCDE model for flower development in lily.

### Floral Organ Identity Genes in Orchids

The structure of the orchid flower has a zygomorphic nature in contrast to most plant groups leading to precise interaction with the pollinator (Cubas, 2004). Orchid flowers generally contain an outer whorl with three sepals, an inner whorl with three petals, and a single column in the center. The sepals in most orchids are enlarged and look like petals. Two of the petals are displayed in a normal shape and one becomes a highly specialized structure called the lip or labellum. The sepals and petals are usually called tepals since they are very similar in appearance. The column, also called the gynostemium, is the reproductive organ of orchids, which combines the gynoecium (female organ) and androecium (male organ). On the top of the column is the male anther which contains packets of pollen called pollinia. Many floral MADS-box genes have been identified from different orchids. According to their expression patterns and putative roles in determining the different floral organs, advanced models of flower development for orchids have been proposed. Also, these studies offer an opportunity to understand the relationships between the diverse array of floral MADS-box genes and flower development.

### A-Class Genes

A- and E-class genes belong to the same *AP1*/*AGL9* superfamily group. A-function genes come from the *SQUA*-like (*SQUAMOSA*: an *Antirrhinum* A-class gene) subgroup, which can be divided into *FUL*/*AGL8*-like and *euAP1*-like clades. E-function genes come from the *SEP*-like subgroup containing *AGL9*-like and *AGL2*/*3*/*4*-like clades. *AGL6* is a member of the *AP1*/*AGL9* group between the *SQUA*-like and *SEP*-like subgroups (Purugganan et al., 1995; Chang et al., 2009). Two *AP1*-like genes, *ORAP11* and *ORAP13*, were isolated and characterized in *Phalaenopsis* Formosa Rose. Both genes are highly expressed during the early stages of floral buds and vegetative organs (Chen et al., 2007). At later stages of flower development, *ORAP11* is expressed only in the column and *ORAP13* expression is absent from all of the floral organs. *PaAP1-1* identified from *P. aphrodite* is expressed in the inner whorls of the pollinia and pedicel whereas *PaAP1-2* is expressed in the pedicel only (Su et al., 2013). This expression pattern suggests that *PaAP1-1* and *PaAP1-2* play a role in the development of the pollinia and gynoecium rather than being involved in the perianth formation like that in *Arabidopsis*. Furthermore, *PaAP2-5* is mainly expressed in whorl 1 and 2, *PaAP2-7* is specifically expressed in pollinia, and *PaAP2-11* is expressed at a low level among all floral organs. Novel functions of homeotic genes may be acquired based on the diversified expression in *Phalaenopsis* through evolutionary processes. Two *AP1/FUL*-like genes in *Phalaenopsis* hybrid “Athens,” *PhaMADS1* and *PhaMADS2* are highly expressed in the ovary before pollination, but a low level of expression is detected in the perianth and gynostemium (Acri-Nunes-Miranda and Mondragon-Palomino, 2014). *DOMADS2* identified from *Dendrobium* Madam Thong-in, and *DthyrFL1*, *DthyrFL2* and *DthyrFL3* from *D. thyrsiflorum* have been characterized as *SQUA*-like and *FUL*-like genes, respectively (Yu and Goh, 2000b; Skipper et al., 2005). The expression of *DOMADS2* could be detected in the SAM during floral transition but it was limited to the column at the later stages of flower development. Low level expression of *DthyrFL1*, *DthyrFL2* and *DthyrFL3* was observed in the vegetative tissues, but higher levels were detected in the inflorescences and ovules. The *AP1* homolog of *Oncidium* Gower Ramsey, *OMADS10* is expressed in vegetative leaves, lip and carpel of mature flowers (Chang et al., 2009). An *AP2*-like gene of *D. crumenatum*, *DcOAP2* is expressed in all floral organs (Xu et al., 2010). Weak expression of three *AP1*-like genes, *EpMADS10*, *EpMADS11* and *EpMADS12* from *Erycina pusilla* has also been observed in all floral organs (Dirks-Mulder et al., 2017). An *AP2-like* gene of *C. ensifolium*, *CeAP2*, is expressed mainly in sepals and petals with negative regulation by miR172 (Yang et al., 2015).

### B-Class Genes

B-class genes in Orchidaceae have been surveyed and have 11 species characterized into four *AP3*- and two *PI*-duplicated homologs (Pan et al., 2011). *PI* homologs are uniformly expressed in all floral whorls whereas different clades of *AP3* homologs may have different expression patterns within floral organs. In *P. equestris*, four *DEF*-like genes, *PeMADS2*, *PeMADS3*, *PeMADS4* and *PeMADS5* have been identified. (Tsai et al., 2004). They are expressed in floral organs with distinct patterns. *PeMADS2* and *PeMADS5* are expressed in sepals, petals, lip and column; *PeMADS3* is expressed in petals, lip and column, whereas *PeMADS4* is expressed in lip and column only. In peloric (lip-like petal) mutant, expression of *PeMADS5* is absent from all floral organs, and expression of *PeMADS4* is extended to the lip-like petal. This indicates that, among the four *DEF*-like genes, *PeMADS4* may determine the lip formation in *Phalaenopsis*. In addition, these four *DEF*-like MADS genes are able to interact with a *GLO*-like gene *PeMADS6* (Tsai et al., 2005; Tsai et al., 2008). Ectopic expression of *PeMADS6* caused petaloid sepals in *Arabidopsis* flowers. Furthermore, these heterodimers could bind the CArG *cis*-element. A single copy of *PI*-like gene of *Phalaenopsis* hybrid, *PhPI10* has been also characterized (Guo et al., 2008). Expression of *PhPI10* is restricted to the lip of flowers. Several *AP3*-like genes *PaAP3-1*, *PaAP3-2*, *PaAP3-3* and *PaAP3-4* were identified in *P. aphrodite* and their expression was preferentially detected in sepals, petals and the lip/column (Su et al., 2013). Another *PI-like* gene, *PaPI*, has also been identified but its transcripts are detectable in all floral organs. In *Dendrobium crumenatum*, *DcOAP3A*/*B* and *DcOPI* were identified as *AP3*-like and *PI*-like genes, respectively (Xu et al., 2006). Transcripts of *DcOAP3A* and *DcOPI* are accumulated in all floral organs and their proteins form heterodimers. Abnormal flowers containing petaloid sepals were produced when *DcOPI* was overexpressed in *Arabidopsis*. In addition, the phenotypic alteration of B-function mutants has also been observed by overexpressing *DcOAP3-SRDX* repressor fusion construct implying a dominant negative effect on the B-function *via* the hetero-dimerization with its interacting partners. The *Oncidium* Gower Ramsey *AP3*-like gene, *OMADS3* is also expressed in all floral organs (Hsu and Yang, 2002). Another study characterized the B-class MADS-box genes of *Oncidium* including the *AP3*-like *OMADS3*, *OMADS5* and *OMADS9*, and *PI*-like *OMADS8* (Chang et al., 2010). *OMADS5* is expressed in sepals and petals; *OMADS9* is expressed in petals and lip; *OMADS8* is expressed in all four floral organs like *OMADS3*. In lip-like petals and lip-like sepals of peloric mutant flowers, expression of *OMADS5* is down-regulated suggesting that *OMADS5* negatively regulates the lip formation. Recently, the perianth formation in *Cymbidium goeringii* was determined through the complete analysis of expression levels of B-class genes along with co-expression of A-class and E-class genes (Xiang et al., 2018). *CgDEF1* is expressed in sepals and petals but not lip; *CgDEF3* and *CgDEF4* are highly expressed in lip and lip-like petals whereas *CgDEF2* is expressed in all floral organs. Several B-class genes have been isolated from *Habenaria* (Kim et al., 2007), *Orchis* (Salemme et al., 2011) and *Erycina* (Dirks-Mulder et al., 2017). *HrDEF* expression is exhibited in the petals and column whereas that of *HrGLO1* and *HrGLO2* is detected in all floral organs. In the petaloid-sepal mutant, expression of *HrDEF* was extended to the petaloid sepals suggesting that distinctive expression of *HrDEF* determines the differentiation of sepals and petals of *H. radiata* flowers. In *Orchis italica*, the *PI*/*GLO*-like genes *OrcPI* and *OrcPI2* are expressed in all floral organs of immature floral buds but their expression is restricted to the lip in the mature flower. In *E. pusilla*, *EpMADS13*, *EpMADS14* and *EpMADS15* are *AP3*-like genes while *EpMADS16* is a *PI*-like gene. Their expression is detectable in almost all floral organs but has diverse patterns. In *Rhynchostylis gigantea*, *RgAP3* an *AP3*-like and *RgPI* a *PI*-like genes were cloned and expression levels of *RgAP3* were noticed only in the petal and sepal, and *RgPI* was expressed in every part of the floral organs (Zhang et al., 2013).

### C- and D-Class Genes

The reproductive organ, the gynostemium, is another unique floral structure in orchids in addition to the labellum. In the ABCDE model, C-class genes are important for the development of stamens and carpels, and D-class genes are required for ovule development. Both C- and D-class genes belong to the *AG* subfamily of MADS-box genes (Theissen et al., 2000; Kramer et al., 2004). In orchids, C- and D-class genes involved in the development of the gynostemium and ovule have been also identified. In *P. equestris*, *PeMADS1* and *PeMADS7* were characterized as C- and D-class genes, respectively (Chen et al., 2012b). Spatial expression analyses of *PeMADS1* and *PeMADS7* demonstrated that they are specifically expressed in the gynostemium of the flower. Development of ovules is initiated after pollination in orchids and *PeMADS1* and *PeMADS7* transcripts are accumulated in the ovules after pollination. Moreover, expression of *PeMADS1* could be detected in petals of *gynostemium-like petal* (*gylp*) mutant suggesting that *PeMADS1* functions in gynostemium development. PeMADS1 and PeMADS7 could form a homodimer or heterodimer *via* the PeMADS8, an E-class protein. Ectopic expression analyses showed that *PeMADS1* could rescue the phenotype of *AG* mutant, and *PeMADS7* in *Arabidopsis* produced characteristic phenotypes of the D-class gene family without homeotic conversions. Another two studies also characterized C- and D-class genes in *Phalaenopsis* Hatsuyuki and *Phalaenopsis* Athens including *AG*-like *PhlAG1*, *PhaMADS8*, *PhaMADS10* and *STK*-like *PhlAG2*, *PhaMADS9* (Song et al., 2006; Acri-Nunes-Miranda and Mondragon-Palomino, 2014). Expressions of *PhlAG1* and *PhlAG2* could be detected in the lip, column and ovule, and *PhaMADS8*, *PhaMADS9* and *PhaMADS10* were specifically expressed in the gynostemium and ovary. Expression of *PhaMADS9* but not *PhaMADS8* and *PhaMADS10* is increased in the gynostemium of peloric mutants suggesting that the expression level of *STK*-like gene is crucial for gynostemium development. The C- and D-class genes in *D. crumenatum* and *D. thyrsiflorum* were also characterized; *DcOAG1* and *DthyrAG1* are C-class genes; *DcOAG2* and *DthyrAG2* are D-class genes (Skipper et al., 2006; Xu et al., 2006). *DcOAG1* is expressed in all floral organs while *DcOAG2* is mainly expressed in the ovary and in the envelope cells of pollinia. Overexpression of *DcOAG1* in *Arabidopsis* transformed the sepals and petals into carpel-like and stamen-like structures, respectively. *DthyrAG1* and *DthyrAG2* are expressed in the inflorescences and ovules after pollination, and *DthyrAG2* is believed to play more important roles in later stages of ovule development in *D. thyrsiflorum* since its expression was higher than that of *DthyrAG1* in ovules. The C- and D-class genes in *Oncidium* Gower Ramsey, namely *OMADS4* and *OMADS2* respectively, were also characterized (Hsu et al., 2010). *OMADS4* is expressed in the stamens and carpels whereas expression of *OMADS2* is restricted to the stigmatic cavity and ovary of carpels. In addition, yeast two-hybrid analyses showed that *OMADS4* and *OMADS2* are able to form homodimers by themselves or form heterodimers with each other. Ectopic expression of *OMADS4* and *OMADS2* caused only early or moderately early flowering in *Arabidopsis* without homeotic conversion of floral organs. C- and D-class genes characterized in *Cymbidium* (Wang et al., 2011b), *Orchis* (Salemme et al., 2013) and *Erycina* (Dirks-Mulder et al., 2017), include C-class genes *CeMADS1*, *CeMADS2*, *OitaAG*, *EpMADS20*, *EpMADS21* and *EpMADS22*, and D-class genes *OitaSTK* and *EpMADS23*. *CeMADS1* is only expressed in the column but *CeMADS2* is expressed in all floral organs. In the multitepal mutant whose male and female reproductive organs are replaced by a newly emerged flower, expression of *CeMADS1* is lost, suggesting that *CeMADS1* is associated with the development of the gynostemium. *O. italica OitaAG* and *OitaSTK* are also expressed in the reproductive organs in both the early and late stages. Among the *E. pusilla* MADS-box genes, *EpMADS20* is expressed in all floral organs while others, such as *EpMADS21*, *EpMADS22* and *EpMADS23* are mainly expressed in the gynostemium.

### E-Class Genes

E-class genes show unique interaction with other floral organ identity genes to determine all floral organs. *Arabidopsis* has four *SEP* genes: *SEP1*, *SEP2*, *SEP3*, and *SEP4*. The flowers of a triple mutant of *sep1 sep2 sep3* consist entirely of sepal-like organs (Pelaz et al., 2000) while the quadruple mutant of *sep1 sep2 sep3 sep4* produces leaf-like organs instead of floral organs (Ditta et al., 2004). Moreover, simultaneously reduced expression of four rice *SEP*-like genes (*OsMADS1*, *OsMADS5*, *OsMADS7* and *OsMADS8*) caused the conversion of all floral organs except the lemma into leaf-like structures (Cui et al., 2010). In orchids, a number of genes belonging to the E-class have been characterized at the molecular level. A *SEP3*-like gene, *OM1* was the first E-class gene identified, from the mature flower of *Aranda* Deborah, and its expression was identified in sepals and petals (Lu et al., 1993). In *Phalaenopsis* hybrid Athens, three *SEP*-like genes *PhaMADS4*, *PhaMADS5* and *PhaMADS7* have been identified and these are expressed in the sepal, petal and labellum (Acri-Nunes-Miranda and Mondragon-Palomino, 2014). In *P. aphrodite*, *PaAGL6-1* is expressed in the lip, whereas the *PaAGL6-2* is expressed throughout all floral organs (Su et al., 2013). Four *SEP*-like genes *PeSEP1*, *PeSEP2*, *PeSEP3* and *PeSEP4* were characterized from *P. equestris* (Pan et al., 2014). These *PeSEP* genes are expressed in all floral organs and VIGS of *PeSEP3* resulted in production of leaf-like tapels. Down-regulation of *PeSEP2* expression alone by VIGS has minor effects, but silencing of both *PeSEP2* and *PeSEP3* expression caused reduced expression of B-class genes such as *PeMADS2*, *PeMADS3*, *PeMADS4*, *PeMADS5* and *PeMADS6* suggesting an association between *PeSEP* functions and B-class gene expression. *DOMADS1* and *DOMADS3* from *Dendrobium* Madame Thong-In are homologous genes of *SEP3* and *SEP4*, respectively, and *DcOSEP1* from *D. crumenatum* is a homolog of *SEP3* (Yu and Goh, 2000b; Xu et al., 2006). Expressions of these genes are constantly activated during floral transition and continue into the mature floral stage. Four E-class genes belonging to the *AP1*/*AGL9* superfamily, *OMADS6*, *OMADS7*, *OMADS10* and *OMADS11* have been identified from *Oncidium* Gower Ramsey (Chang et al., 2009). *OMADS6* is expressed in all floral organs except the stamen, and expression patterns of *OMADS7* and *OMADS11* are similar to that of *OMADS6*. Unlike *OMADS6*, *OMADS10* is expressed in vegetative leaves although its transcripts are specifically accumulated in the lip and carpels of mature flowers. Moreover, overexpression of *OMADS6*, *OMADS7* and *OMADS11* resulted in extremely early flowering but *OMADS10* overexpression caused moderately early flowering in *Arabidopsis*. Several *AGL6*-like genes including *EpMADS3*, *EpMADS4* and *EpMADS5* have been identified in *E. pusilla*. They are expressed in floral organs with various expression levels in distinct floral organs suggesting that multiple *AGL6*-like genes may also contribute to the development of floral organs (Dirks-Mulder et al., 2017). Seven E-class genes *CgSEP1*, *2*, *3*, *4* and *CgAGL6-1, -2, -3* were characterized in *C. goeringii* (Xiang et al., 2018). The expression level of *CgSEP1* was increased in the peloric mutant lips and decreased in the peloric mutant sepals. High expression levels of *CgSEP2* and *CgAGL6-1* could be detected in the sepals, but rarely in the lips and columns of the wild-type and the peloric mutant. An increased expression level of *CgSEP3* was noticed in peloric mutant lip-like petals and lips, but expression level of *CgSEP4* was decreased in the peloric mutant lip-like petals and lips. The expression level of *CgAGL6-2* is increased in all floral organs of the wild-type and peloric mutant, but increased level of *CgAGL6-3* could only be detected the in lip and lip-like petals of wild-type and peloric mutant. Based on expression patterns and phenotypic alterations caused by the E-class genes, it is likely that E-class genes are required for petal, stamen, and carpel formation in both dicot and monocot plants. Of note, a recent report has shown that the greenish flower phenotype of a mutant orchid cultivar in *Habenaria radiata* is due to the absence of SEP (*HrSEP-1*) function (Mitoma and Kanno, 2018). The characterized genes of flower development in orchids are listed in **Table S2** (Aceto and Gaudio, 2011; Mondragon-Palomino, 2013; Teixeira da Silva et al., 2014).

### Connective Codes for Perianth Formation

Genetic models related to the regulatory patterning formation of actinomorphic flowers in *Arabidopsis* and *Antirrhinum* are well characterized. However, orchid flowers usually have zygomorphic symmetry with a prominent, well-differentiated labellum in the inner tepals, and it is morphologically distinct from tepals of other angiosperms (Salemme et al., 2011). The perianth formation in orchids cannot be solely explained by the ABCDE model. Many divergent genes with novel functions are responsible for the regulation of perianth formation in orchids. The model “Orchid Code” has been proposed to explain how the various expression levels of duplicated *AP3*/*DEF*-like genes regulate the perianth morphogenesis in orchids (**Figure 2B**) (Mondragon-Palomino and Theissen, 2008; Mondragon-Palomino and Theissen, 2009). In this model, duplicated *DEF*-like paralogous are grouped into four clades phylogenetically and their expression patterns determine the formation of different flower organs. The development of outer tepals (sepal) is regulated by the co-expression of clade 1 (*PeMADS2*-like) and clade 2 (*PeMADS5-like* or *OMADS3*-like) genes whereas the two lateral petals are regulated by the co-expression of clade 1, clade 2 and clade 3 (*PeMADS3*-like) genes. The lip structure is regulated by the co-expression of clade 1, clade 2, clade 3 and clade 4 (*PeMADS4*-like) genes. In contrast, *GLO*-like genes form a single clade and express in all four floral whorls. In other words, the divergent *DEF*-like gene is the key that leads to the morphological diversity of the flowers in orchids. Later, the orchid code model was refined by extensive analysis of *DEF*-like genes in different subfamilies of orchids (Mondragon-Palomino and Theissen, 2011). According to this refined orchid code, four clades of *DEF*-like genes are all expressed in lateral petal and lip. However, lower expression level of clade 3 and clade 4 is displayed in lateral petal whereas lower expression level of clade 1 and clade 2 genes is displayed in the lip. Moreover, a combination of four clade genes with differential expression level may also contribute to the developments of gynostemium and ovary.

Development of different floral organs of orchids has also been proposed by a homeotic orchid tepal (HOT) model (Pan et al., 2011). Twenty-four *AP3*-like genes were identified from 11 species of orchids and grouped into four clades (*PeMADS3*-like, *PeMADS4*-like, *PeMADS2*-like and *PeMADS5*-like). Similar to the orchid code model, the sepal organ expresses *PeMADS2*-like and *PeMADS5*-like genes whereas the lateral petal expresses *PeMADS2*-like, *PeMADS5*-like and *PeMADS3*-like genes. In the lip organ, all four clade genes are expressed. In addition, the HOT model also addresses the temporal change of *PeMADS3*-like and *PeMADS4*-like expressions in different stages of inflorescence development. In the early floral organ primordial stage, they are expressed in all whorl organs. In the late floral organ primordial and floral bud stages, *PeMADS3*-like genes are restricted in petal, lip and column whereas the *PeMADS4*-like genes are restricted in the lip and column. Combinations of *PI*-like genes and other MADS-box genes also determine the identity of lip and column.

Another study also based on the expression analysis proposed a “Perianth code” (P code) hypothesis to explain the floral identity of orchids (**Figure 2B**) (Hsu et al., 2015). Two clades of *AP3*-like genes (*OAP3-1* and *AP3-2*) in *Oncidium* associate with *OPI* and different clades of O*AGL6*-like genes (E-class). Different heterotetradmers determine different organs. The SP (sepal/petal) complex (*OAP3-1*/*OAGL6-1*/*OAGL6-1*/*OPI*) specifies the sepal/petal formation whereas the L (lip) complex (*OAP3-2*/*OAGL6-2*/*OAGL6-2*/*OPI*) is exclusively required for lip formation. The P code model suggests that the diverse E-class genes are also important for development of lip structure of orchids. Diverse roles of E-class genes in floral identity have also been suggested in lily (Tzeng et al., 2003). Two *AGL2*-like genes, *LMADS3* and *LMADS4*, were identified in lily, and they were expressed in all four whorls of floral organs. Ectopic expression of *LMADS3* but bot *LMADS4* in *Arabidopsis* resulted in reduced plant size, early flowering and loss of floral determinacy. In *H. radiata*, two E-class genes (*HrSEP-1* and *HeSEP-2*) were characterized recently (Mitoma and Kanno, 2018). The expression of *HrSEP-1* is lost in a mutant cultivar “Ryokusei” that has greenish petals and a smaller lip in whorl 2, and several septaloid organs and a ventral column in whorls 3 and 4. Moreover, the expression of *HrSEP-2* is up-regulated in Ryokusei. Alternative expressions of B-class genes by silencing of E-class genes have also been revealed in *Phalaenopsis* (Pan et al., 2014). This indicates a feedback regulation between the floral identity genes may also play an important role in the development of floral organs.

All these models specify that the diverse roles of duplicated *AP3*/*DEF*-like and other floral identify genes are important for specialized flower morphogenesis, especially for the lip development of orchid flowers. This idea is supported by the recent study of whole-genome sequencing of *Apostasia shenzhenica*, a genus of Apostasioideae, which is regarded as being phylogenetically plesiomorphic in the orchid family (Zhang et al., 2017). The *Apostasia* has a radially symmetrical (actinomorphic) flower without lip and complex column structures. Comparing *A. shenzhenica* to other subfamilies of Orchidaceae, *A. shenzhenica* seems to have fewer B-class *AP3* genes.

### Orchid Biotechnology

Conventional breeding efforts were initiated more than one century ago and helped propel the growth of the ornamental orchid market. In order to obtain profitable horticultural traits, successful breeding usually proceeds by preserving or selecting advantageous parents or progenies. Application of a well-controlled cultivation management system is another important factor for successful commercial orchid farming. In the worldwide market, hundreds and thousands of successful cultivars have been generated using the conventional methods through enthusiastic professional and amateur breeders. The discovery of native tetraploid *Phalaenopsis* orchids that have better flower shapes and color intensities initiated the creation of numerous significant commercial hybrids through polyploidy breeding. Over the last two decades, a successful program to convert 20 diploid *Phalaenopsis* species to tetraploid has been developed by Chen et al. (2011). This program facilitated a breeding process that feeds the needs of the nursery business. Polyploidization can typically be achieved by introduction of colchicine in many agricultural or horticultural crops including orchids (Griesbach, 1981; Caperta et al., 2006; Azmi et al., 2016; Tuwo and Indrianto, 2016). One of the most successful product series, “Big White Flower,” is comprised of tetraploid *Phalaenopsis* hybrids developed through polyploidization with the parents *Phalaenopsis* Doris. Among them, *P*. Sogo Yukidian ‘V3’ has been very popular in the market. At present, it is still one of the most profitable hybrids in this group. Chen et al. concluded that the importance of polyploidy to the improvement of orchid cultivars may rely on the increase in the number of sets of genes (Chen et al., 2011). The accumulation of additive genetic and heterotic effects of these genes could potentially improve the varieties possible. Currently, polyploidy is still a major technique that drives orchid breeding programs to commercial success. An alternative strategy for polyploid breeding is the phenomenon of unreduced gametes. By analyzing sporad types, Bolaños-Villegas et al. observed a certain percentage of dyads (2n gametes) and suggested the pollination from individual of these dyad may produce polyploid progeny as revealed in other plants (Bolaños-Villegas et al., 2008).

In the 1970s, the creation of recombination DNA molecules brought about revolutionary genetic engineering, also called genetic transformation, which constitutes the direct manipulation of an organism’s genome using biotechnology. It is a set of technologies that is used to change genetic makeup, including the transfer of genes within and across species boundaries to produce improved or novel traits. In 2013, the blue *Phalaenopsis* orchids created by the research team of Professor Mii at Chiba University, Japan were exhibited at the 11th Asian Pacific Orchid Conference. These true-blue orchids were genetically transformed using a flavonoid 3’,5’-hydroxylase gene derived from *Commelina communis* which was incorporated into the *Phalaenopsis* genome and expressed in the flowers to produce delphinidin, a blue anthocyanin pigment that is also in the flowers of delphinium (larkspur). In 2016, the white *Oncidium* orchids created by a research group from the National Taiwan University, Taiwan were exhibited at the Taiwan International Orchid Show. The genetically engineered white orchids were transformed by a flower specific promotor driving the carotenoid cleavage dioxygenase gene that degrades carotenoids, which led to white petals.

Similar to many other crops, two major transformation systems were successfully used to transport foreign genes into orchid genomes: particle bombardment and *Agrobacterium*-mediated transformation systems. The early genetic transformation studies in orchids were confined to the biolistic-mediated transformation which needed a so-called gene gun to deliver the target genes (Kuehnle and Sugii, 1992; Chia et al., 1994). The first successful genetic transformation reported using the *Agrobacterium*-mediated method was the transfer of *gus* gene into *Phalaenopsis* orchid (Belarmino and Mii, 2000). Many years later, this system also successfully transformed other genera of orchids such as *Dendrobium*, *Cymbidium* and *Oncidium* (Yu et al., 2001; Liau et al., 2003; Chin et al., 2007). Target explants for transformation using protocorms, protocorm-like bodies (PLB) and calluses have been reported so far (Belarmino and Mii, 2000; Mishiba et al., 2005; Chin et al., 2007). Usually, the genetic transformation efficiency has been low for orchid plants and the transformation process is also time-consuming. The general slow growth rate could mean the entire transformation process could take up to 2 or 3 years to reach the stage of two-leaf young plantlets. Recently, a protocol using protocorms that could shorten the transformation process to 8 months was reported for *Phalaenopsis* orchids (Hsing et al., 2016). It will be worth investigating whether a similar time frame can be achieved in other orchid genera. Genome editing, a new and revolutionary genetic engineering technology, is an approach in which a specific target DNA sequence of the genome is altered by adding, removing, or replacing DNA bases in a highly precise manner (Gaj et al., 2013). One particular tool, the CRISPR/Cas system has been developed at an accelerated pace. The application of the CRISPR/Cas system to create engineered crops or plants has yielded fruitful results for further investigation. The technology development and application have been widely reviewed (Bortesi and Fischer, 2015; Yin et al., 2017). One crucial criterion to precisely edit orchid genomes is to rely heavily on the availability of the whole genome sequence information. We predict that the combination of the genome editing technology with bioinformatics analyses will create revolutionary breeding programs for orchid research and development in the future.

## Perspectives

For annual plants such as *Arabidopsis*, rice and wheat, precise control of flowering time in response to environmental cues is necessary for successful reproduction. For perennial plants like orchids, the flowering network may not be so complicated since vernalization is not required and the effect of photoperiod is limited. The changes in ambient temperature may play a more important role in the regulation of floral transition in orchids. Recently, regulations of microRNAs and anti-florigens such as *TFL1* have been shown to be involved in the flowering control of *A. alpina*, a perennial relative of *A. thaliana* (Wang et al., 2011a; Bergonzi et al., 2013). Although further investigation is required to discover whether orchid *TFL1* also plays a negative role in flowering in orchids, it is no exaggeration to say that *TFL1* may regulate the dormancy of juvenile axillary buds in the flowering season of orchids. Furthermore, regulation of *PHYTOCHROME INTERACTING FACTOR 4* (*PIF4*) *via* phytohormone pathways and sugar metabolism may also be involved (Kumar et al., 2012; Bolouri Moghaddam and Van den Ende, 2013). Flower development studies revealed that multiplicated floral homeotic genes (**Table S2**) are the basis of the specific morphology of orchids (**Figure 2**). However, how the high order complex of these proteins regulates floral organ identity needs to be further investigated. In addition, the feedback regulation between different floral homeotic genes needs further investigation to explain the floral development of orchids in detail. The A and E-class genes are involved in both floral initiation and flower development. It will be interesting to investigate whether A and E-class genes regulate the expressions of other MADS-box genes. Finally, further advances in functional studies on key genes for flowering/flower development may rely on a breakthrough in orchid transformation technology which leads to more efficient results. Recently, the genome sequences of several orchids have been determined. Understanding of the flowering control and flower development of the CAM-using plants may provide an insightful view into plant evolution.

## Author Contributions

All the authors wrote and reviewed the manuscript.

## Funding

This work was supported in part by grants from World Vegetable Center Korea Office (WKO #10000379) and core donors to the World Vegetable Center: Republic of China (ROC), UK aid from the UK government, United States Agency for International Development (USAID), Australian Center for International Agricultural Research (ACIAR), Germany, Thailand, Philippines, Korea and Japan and, a grant from the Ministry of Science and Technology, Taiwan (MOST 106-2321-B-020-002-) to F-C. C. and a grant from Council of Agriculture, Taiwan (COA 106AS-8.6.3-FD-Z1(1)) to F-C. C.
