# Supplementary material for: Floral Induction and Flower Development of Orchids
Source: Front Plant Sci. 2019 Oct 10;10:1258. doi: 10.3389/fpls.2019.01258 (PMC6795766; doi:10.3389/fpls.2019.01258)
Supplement: Supplementary file 1 [file Table_1.docx]

Table S1. The putative flowering genes in three orchids.

| *Arabidopsis* | *Phalaenopsis* |  |  | *Dendrobium* |  |  | *Oncidium* |  |  |
| --- | --- | --- | --- | --- | --- | --- | --- | --- | --- |
|  | contig ID | amino acid aligned | identity (%) | contig ID | amino acid aligned | identity (%) | contig ID | amino acid aligned | identity (%) |
| *FT* | PATC022841 | 131 | 58.02 | DNTC004082 | 67 | 55.22 | OGTC046025 | 128 | 59.38 |
| *TFL1* | PATC134478 | 125 | 68.00 | DNTC002178 | 125 | 66.40 | Not detected |  |  |
| *ATC* | PATC022841 | 170 | 55.88 | DNTC002178 | 168 | 48.81 | OGTC046025 | 170 | 55.88 |
| *FD* | PATC134641 | 225 | 37.33 | DNTC011762 | 77 | 67.53 | OGTC041187 | 67 | 71.64 |
| *LFY* | PATC112706 | 161 | 87.58 | Not detected |  |  | OGTC022784 | 158 | 86.71 |
| *AP1* | PATC201550 | 192 | 65.62 | DNTC013820 | 192 | 66.67 | OGTC046039 | 223 | 56.95 |
| *SOC1* | PATC154491 | 216 | 52.31 | DNTC005124 | 64 | 79.69 | OGTC017221 | 218 | 49.54 |
| *SVP* | PATC127095 | 88 | 71.59 | DNTC002694 | 88 | 79.55 | OGTC009658 | 187 | 72.41 |
| *FLM* | PATC141899 | 71 | 54.93 | DNTC012661 | 55 | 60.00 | OGTC046040 | 55 | 63.64 |
| *CO* | PATC156501 | 88 | 56.82 | DNTC013733 | 79 | 62.03 | OGTC012733 | 79 | 62.03 |
| *FKF1* | PATC148381 | 186 | 75.00 | DNTC001923 | 77 | 65.81 | OGTC044830 | 174 | 70.16 |
| *GI* | PATC212608 | 143 | 66.20 | Not detected |  |  | OGTC027539 | 216 | 83.72 |
| *CDF1* | PATC150032 | 57 | 83.82 | DNTC003893 | 57 | 59.38 | OGTC045129 | 57 | 78.08 |
| *PIF4* | PATC132654 | 155 | 52.26 | Not detected |  |  | OGTC022318 | 83 | 66.27 |
| *DhELF2*#([Chen et al., 2015](#_ENREF_22)) | PATC143251 | 131 | 100 | DNTC001133 | 111 | 81.08 | OGTC001004 | 113 | 92.04 |
| *DOH1* (Yu et al., 2000)# | PATC145786 | 74 | 90.54 | DNTC013818 | 65 | 95.38 | OGTC044820 | 74 | 91.89 |
| *DOMADS1* (Yu et al., 2000)# | PATC141808 | 170 | 80.58 | DNTC013812 | 174 | 89.66 | OGTC046044 | 171 | 77.78 |

The homologs of different flowering-related genes in *Phalaenopsis aphrodite*, *Dendrobium nobile* and *Oncdium* Grower Ramsey. The results were obtained by using the amino acid sequences of *Arabidopsis* genes for BLAST search against the transcriptome database Orchidstra 2.0. (http://orchidstra2.abrc.sinica.edu.tw/orchidstra2/index.php; Chao et al., 2017). #The published orchids genes were used for the BLAST search.
