# Supplementary material for: Floral Induction and Flower Development of Orchids
Source: Front Plant Sci. 2019 Oct 10;10:1258. doi: 10.3389/fpls.2019.01258 (PMC6795766; doi:10.3389/fpls.2019.01258)
Supplement: Supplementary file 2 [file Table_2.docx]

Table S2. Characterized A, B, C, D, E-class genes in orchids^§^.

| Class | Gene | GenBank Accession Number | Species | Clade | Expression Pattern  S P L C P/O VT Ovules | | | | | | |
| --- | --- | --- | --- | --- | --- | --- | --- | --- | --- | --- | --- |
| **A** | *DOMADS2* | AF198175 | *Dendrobium* Madame Thong-In | *AP1*/*FUL*-like | **-** | **-** | **-** | **+** | - | +^c^ | ND |
|  | *DthyrFL1* | AY927236 | *Dendrobium thyrsiﬂorum* | *AP1*/*FUL*-like | ND | ND | ND | ND | ND | + | + |
|  | *DthyrFL2* | AY927237 | *Dendrobium thyrsiﬂorum* | *AP1*/*FUL*-like | ND | ND | ND | ND | ND | + | + |
|  | *DthyrFL3* | AY927238 | *Dendrobium thyrsiﬂorum* | *AP1*/*FUL*-like | ND | ND | ND | ND | ND | + | + |
|  | *OMADS10* | HM140846 | *Oncidium* Gower Ramsey | *AP1*/*AGL9*-like | ND | ND | + | + | ND | +^b^ | ND |
|  | *ORAP11* | DQ104328 | *Phalaenopsis* Formosa Rose | *AP1*/*FUL*-like | - | - | - | + | - | ND | ND |
|  | *ORAP13* | DQ104327 | *Phalaenopsis* Formosa Rose | *AP1*/*FUL*-like | - | - | - | - | - | ND | ND |
|  | *EpMADS10* | KJ002735 | *Erycina pusilla* | *AP1*/*FUL*-like | + | + | + | + | + | ND | ND |
|  | *EpMADS11* | KJ002736 | *Erycina pusilla* | *AP1*/*FUL*-like | + | + | + | + | + | ND | ND |
|  | *EpMADS12* | KJ002737 | *Erycina pusilla* | *AP1*/*FUL*-like | + | + | + | + | + | ND | ND |
|  | *DcOAP2* | DQ119837 | *Dendrobium crumenatum* | *AP2*-like | + | + | + | + | + | ND | ND |
|  | *PaAP1-1* | ^©^PATC145405 | *Phalaenopsis aphrodite* | *AP1*/*FUL*-like | - | - | - | - | + | ND | ND |
|  | *PaAP1-2* | ^©^PATC154931 | *Phalaenopsis aphrodite* | *AP1*/*FUL*-like | - | - | - | - | + | ND | ND |
|  | *PaAP2-5* | ^©^PATC133172 | *Phalaenopsis aphrodite* | *AP2*-like | + | + | - | - | - | ND | ND |
|  | *PaAP2-7* | ^©^PATC138345 | *Phalaenopsis aphrodite* | *AP2*-like | - | - | - | - | + | ND | ND |
|  | *PaAP2-11* | ^©^PATC124448 | *Phalaenopsis aphrodite* | *AP2*-like | + | + | + | + | + | ND | ND |
|  | *PhaMADS1* | Not available | *Phalaenopsis* hybrid “Athens”, | *AP1*/*FUL*-like | - | - | - | - | + | +^b^ | ND |
|  | *PhaMADS2* | Not available | *Phalaenopsis* hybrid “Athens”, | *AP1*/*FUL*-like | - | - | - | - | + | +^b^ | ND |
|  | *CgAP1-1* | KX347440 | *Cymbidium goeringii* | *AP1*/*FUL*-like | + | + | + | + | ND | ND | ND |
|  | *CgAP1-2* | KX347441 | *Cymbidium goeringii* | *AP1*/*FUL*-like | + | + | + | + | ND | ND | ND |
|  | *CgAP1-3* | KX347442 | *Cymbidium goeringii* | *AP1*/*FUL*-like | - | + | + | + | ND | ND | ND |
|  | *CgAP2-1* | KX347443 | *Cymbidium goeringii* | *AP2*-like | + | + | + | + | ND | ND | ND |
|  | *CgAP2-2* | KX347444 | *Cymbidium goeringii* | *AP2*-like | + | + | + | + | ND | ND | ND |
|  | *CgAP2-3* | KX34745 | *Cymbidium goeringii* | *AP2*-like | + | + | + | + | ND | ND | ND |
|  | *CeAP2* | Not available | *Cymbidium ensifolium* | *AP2*-like | + | + | - | + | ND | ND | ND |
| **B** | *DcOAP3A* | DQ119838 | *Dendrobium crumenatum* | *AP3*/*DEF*-like | + | + | + | + | + | +^b^ | ND |
|  | *DcOAP3B* | DQ119839 | *Dendrobium crumenatum* | *AP3*/*DEF*-like | - | + | + | + | + | ND | ND |
|  | *DMMADS4* | GU132995 | *Dendrobium moniliforme* | *AP3*/*DEF*-like | - | + | + | + | - | ND | + |
|  | *DMAP3A* | EU056327 | *Dendrobium moniliforme* | *AP3*/*DEF*-like | + | + | + | + | + | +^b^ | + |
|  | *DMAP3B* | EU056328 | *Dendrobium moniliforme* | *AP3*/*DEF*-like | - | + | + | - | - | - | - |
|  | *OMADS3* | AY196350 | *Oncidium* Gower Ramsey | *AP3*/*DEF*-like | + | + | + | + | + | +^b^ | + |
|  | *OMADS5* | HM140840 | *Oncidium* Gower Ramsey | *AP3*/*DEF*-like | + | + | - | - | - | - | - |
|  | *OMADS9* | HM140841 | *Oncidium* Gower Ramsey | *AP3*/*DEF*-like | - | + | + | - | - | - | - |
|  | *OMADS12* | Not available | *Oncidium* Gower Ramsey | *AP3*/*DEF*-like | - | - | - | + | ND | ND | ND |
|  | *HrDEF* | AB232663 | *Habenaria radiata* | *AP3*/*DEF*-like | - | + | + | + | - | - | - |
|  | *PeMADS2* | AY378149 | *Phalaenopsis equestris* | *AP3*/*DEF*-like | + | + | - | - | - | - | ND |
|  | *PeMADS3* | AY378150 | *Phalaenopsis equestris* | *AP3*/*DEF*-like | - | + | + | - | - | - | ND |
|  | *PeMADS4* | AY378147 | *Phalaenopsis equestris* | *AP3*/*DEF*-like | - | - | + | + | - | - | ND |
|  | *PeMADS5* | AY378148 | *Phalaenopsis equestris* | *AP3*/*DEF*-like | + | + | + | - | - | - | ND |
|  | *PhlonDEF2* | FJ804106 | *Phragmipedium longiflorum* | *AP3*/*DEF*-like | + | + | - | + | - | ND | ND |
|  | *PhlonDEF1* | FJ804105 | *Phragmipedium longiflorum* | *AP3*/*DEF*-like | + | + | - | + | + | ND | + |
|  | *PhlonDEF3* | FJ804107 | *Phragmipedium longiflorum* | *AP3*/*DEF*-like | - | + | + | + | - | ND | - |
|  | *PhlonDEF4* | FJ804108 | *Phragmipedium longiflorum* | *AP3*/*DEF*-like | - | + | + | + | - | ND | - |
|  | *VaplaDEF1* | FJ804115 | *Vanilla planifolia* | *AP3*/*DEF*-like | + | + | - | + | - | ND | ND |
|  | *VaplaDEF2* | FJ804116 | *Vanilla planifolia* | *AP3*/*DEF*-like | - | + | + | + | - | ND | ND |
|  | *VaplaDEF3* | FJ804117 | *Vanilla planifolia* | *AP3*/*DEF*-like | - | + | + | + | + | ND | + |
|  | *PaAP3-1* | ^©^PATC240636 | *Phalaenopsis aphrodite* | *AP3*/*DEF*-like | + | + | - | - | - | ND | - |
|  | *PaAP3-2* | ^©^PATC133864 | *Phalaenopsis aphrodite* | *AP3*/*DEF*-like | + | + | - | - | - | ND | ND |
|  | *PaAP3-3* | ^©^PATC138350 | *Phalaenopsis aphrodite* | *AP3*/*DEF*-like | - | + | + | + | - | ND | _ |
|  | *PaAP3-4* | ^©^PATC154853 | *Phalaenopsis aphrodite* | *AP3*/*DEF*-like | - | + | + | + | - | ND | - |
|  | *CgDEF1* | HM106983 | *Cymbidium goeringii* | *AP3*/*DEF*-like | + | + | - | - | - | ND | ND |
|  | *CgDEF2* | KU058678 | *Cymbidium goeringii* | *AP3*/*DEF*-like | + | + | + | + | + | ND | ND |
|  | *CgDEF3* | HM106982 | *Cymbidium goeringii* | *AP3*/*DEF*-like | - | + | + | - | - | ND | ND |
|  | *CgDEF4* | KX347446 | *Cymbidium goeringii* | *AP3*/*DEF*-like | - | + | + | - | - | ND | ND |
|  | *BnAP3-1* | EU444021 | *Brassavola nodosa* | *AP3*/*DEF*-like | + | - | - | - | ND | ND | ND |
|  | *EpMADS13* | KJ002738 | *Erycina pusilla* | *AP3*/*DEF*-like | + | + | + | - | ND | ND | ND |
|  | *EpMADS14* | KJ002739 | *Erycina pusilla* | *AP3*/*DEF*-like | + | - | - | - | ND | ND | ND |
|  | *EpMADS15* | KJ002740 | *Erycina pusilla* | *AP3*/*DEF*-like | + | + | - | - | ND | ND | ND |
|  | *DenAP3-1* | EU444025 | *Dendrobium* Spring Jewel | *AP3*/*DEF*-like | + | + | + | + | - | ND | ND |
|  | *DenAP3-2* | EU444026 | *Dendrobium* Spring Jewel | *AP3*/*DEF*-like | - | + | + | + | - | ND | ND |
|  | *DenAP3-3* | EU444027 | *Dendrobium* Spring Jewel | *AP3*/*DEF*-like | - | - | + | - | - | ND | ND |
|  | *AfAP3-1* | EU444018 | *Anoectochilus formosanus* | *AP3*/*DEF*-like | - | + | + | + | - | ND | ND |
|  | *PaphAP3-1* | EU444046 | *Paphiopedilum* Macabre | *AP3*/*DEF*-like | - | - | + | + | - | ND | ND |
|  | *PaphAP3-2* | EU444047 | *Paphiopedilum* Macabre | *AP3*/*DEF*-like | - | + | + | + | - | ND | ND |
|  | *PaphAP3-3* | EU444048 | *Paphiopedilum* Macabre | *AP3*/*DEF*-like | + | + | + | + | - | ND | ND |
|  | *LdAP3-1* | EU444036 | *Liparis distans* | *AP3*/*DEF*-like | + | + | - | + | - | ND | ND |
|  | *DcOPI* | Not available | *Dendrobium crumenatum* | *PI*/*GLO*-like | + | + | + | + | + | ND | ND |
|  | *DMPI* | EU056326 | *Dendrobium moniliforme* | *PI*/*GLO*-like | + | + | + | + | + | ND | + |
|  | *OMADS8* | HM140842 | *Oncidium* Gower Ramsey | *PI*/*GLO*-like | + | + | + | - | - | +^ab^ | - |
|  | *PhPI15* | AY771992 | *Phalaenopsis hybrid* | *PI*/*GLO*-like | + | + | + | + | + | - | + |
|  | *HrGLO1* | AB232665 | *Habenaria radiata* | *PI*/*GLO*-like | + | + | + | + | - | - | - |
|  | *HrGLO2* | AB232664 | *Habenaria radiata* | *PI*/*GLO*-like | - | + | + | - | - | - | - |
|  | *OrcPI* | AB094985 | *Orchis italica* | *PI*/*GLO*-like | - | - | + | - | - | ND | ND |
|  | *OrcP2* | AB537504 | *Orchis italica* | *PI*/*GLO*-like | - | + | + | - | - | ND | ND |
|  | *PeMADS6* | AY678299 | *Phalaenopsis equestris* | *PI*/*GLO*-like | + | + | + | + | + | - | ND |
|  | *PhlonGLO1* | FJ804109 | *Phragmipedium longiflorum* | *PI*/*GLO*-like | + | + | + | + | + | ND | + |
|  | *VaplaGLO1* | FJ804118 | *Vanilla planifolia* | *PI*/*GLO*-like | + | + | + | + | + | ND | + |
|  | *PaPI* | ^©^PATC152852 | *Phalaenopsis aphrodite* | *PI*/*GLO*-like | + | + | + | + | + | ND | + |
|  | *CgGLO* | HM106984 | *Cymbidium goeringii* | *PI*/*GLO*-like | + | + | + | + | + | ND | ND |
|  | *EpMADS16* | KJ002741 | *Erycina pusilla* | *PI*/*GLO*-like | + | + | + | + | ND | ND | ND |
|  | *PaphPI* | EU444049 | *Paphiopedilum* Macabre | *PI*/*GLO*-like | + | + | + | + | - | ND | ND |
|  | *LdPI* | EU444038 | *Liparis distans* | *PI*/*GLO*-like | + | + | + | + | - | ND | ND |
|  | *PtPI* | EU444053 | *Phaius tankervilleae* | *PI*/*GLO*-like | + | + | + | + | - | ND | ND |
| **C** | *PhalAG1* | AB232952 | *Phalaenopsis* Hatsuyuki | *AG*-like | - | - | - | + | + | ND | + |
|  | *OMADS4* | KJ819939 | *Oncidium* Gower Ramsey | *AG*-like | - | - | - | + | + | - | ND |
|  | *CeMADS1* | GU123626 | *Cymbidium ensifolium* | *AG*-like | - | - | - | + | - | ND | ND |
|  | *CeMADS2* | GU123627 | *Cymbidium ensifolium* | *AG*-like | + | + | + | + | + | ND | + |
|  | *DthyrAG1* | DQ017702 | *Dendrobium thyrsiflorum* | *AG*-like | - | - | - | + | ND | ND | + |
|  | *PeMADS1* | AF234617 | *Phalaenopsis equestris* | *AG*-like | - | - | - | + | + | - | + |
|  | *DcOAG1* | DQ119840 | *Dendrobium crumenatum* | *AG*-like | + | + | + | + | + | ND | ND |
|  | *EpMADS20* | KJ002745 | *Erycina pusilla* | *AG*-like | + | + | + | + | ND | ND | ND |
|  | *EpMADS21* | KJ002746 | *Erycina pusilla* | *AG*-like | - | - | - | + | ND | ND | ND |
|  | *EpMADS22* | KJ002747 | *Erycina pusilla* | *AG*-like | - | - | - | + | ND | ND | ND |
|  | *OitaAG* | JX205496 | *Orchis italica* | *AG*-like | - | - | - | + | + | ND | ND |
|  | *PhaMADS8* | Not available | *Phalaenopsis* hybrid “Athens”, | *AG*-like | - | - | - | + | + | ND | - |
|  | *PhaMADS10* | Not available | *Phalaenopsis* hybrid “Athens”, | *AG*-like | - | - | - | + | + | ND | - |
|  | *PaAG-1* | ^©^PATC052371 | *Phalaenopsis aphrodite* | *AG*-like | - | - | - | + | + | ND | ND |
| **D** | *DcOAG2* | DQ119841 | *Dendrobium crumenatum* | *STK*-like | - | - | - | + | + | ND | ND |
|  | *DthyrAG2* | DQ017703 | *Dendrobium thyrsiflorum* | *STK*-like | - | - | - | + | ND | ND | + |
|  | *PhalAG2* | AB232953 | *Phalaenopsis* Hatsuyuki | *STK*-like | - | - | - | + | + | ND | + |
|  | *OMADS2* | KJ819938 | *Oncidium* Gower Ramsey | *STK*-like | _ | - | - | + | + | - | ND |
|  | *PeMADS7* | JN983500 | *Phalaenopsis equestris* | *STK*-like | - | - | - | + | + | - | + |
|  | *EpMADS23* | KJ002748 | *Erycina pusilla* | *STK*-like | - | - | - | + | ND | ND | ND |
|  | *PaAG-4* | ^©^PATC202120 | *Phalaenopsis aphrodite* | *STK*-like | - | - | - | - | + | ND | ND |
|  | *OitaSTK* | JX205499 | *Orchis italica* | *STK*-like | - | - | - | + | + | ND | + |
|  | *PhaMADS9* | Not available | *Phalaenopsis hybrid “Athens”,* | *STK*-like | - | - | - | + | + | - | + |
| **E** | *AdOM1* | Not available | *Aranda* Deborah | *SEP3*-like | + | + | + | - | ND | ND | ND |
|  | *DcOSEP1* | DQ119842 | *Dendrobium crumenatum* | *SEP1/2*/*4*-like | + | + | + | + | + | - | ND |
|  | *DOMADS1* | AF198174 | *Dendrobium* Madame Thong-In | *SEP1/2*/*4*-like | + | + | + | + | + | - | ND |
|  | *DOMADS3* | AF198176 | *Dendrobium* Madame Thong-In | *SEP3*-like | - | - | - | - | + | - | - |
|  | *OMADS6* | HM140844 | *Oncidium* Gower Ramsey | *SEP3*-like | + | + | + | + | - | - | ND |
|  | *OMADS11* | HM140847 | *Oncidium* Gower Ramsey | *SEP1*/*2*/*4*-like | + | + | + | + | - | ND | ND |
|  | *OMADS1* | HM140843 | *Oncidium* Gower Ramsey | *AGL6*-like | - | - | + | + | - | - | ND |
|  | *OMADS7* | HM140845 | *Oncidium* Gower Ramsey | *AGL6*-like | + | + | + | + | - | ND | ND |
|  | *PhaMADS4* | Not available | *Phalaenopsis* hybrid “Athens”, | *SEP1*/*2*/*4*-like | + | + | + | + | + | ND | + |
|  | *PhaMADS5* | Not available | *Phalaenopsis* hybrid “Athens”, | *SEP3*-like | + | + | + | + | + | +^a,b^ | + |
|  | *PhaMADS7* | Not available | *Phalaenopsis* hybrid “Athens”, | *SEP3*-like | + | + | + | + | + | +^a^ | + |
|  | *PeSEP1* | KF673857 | *Phalaenopsis equestris* | *SEP3*-like | + | + | + | + | + | - | + |
|  | *PeSEP2* | KF673858 | *Phalaenopsis equestris* | *SEP1*/*2*/*4*-like | + | + | + | + | + | +^b^ | + |
|  | *PeSEP3* | KF673859 | *Phalaenopsis equestris* | *SEP3*-like | + | + | + | + | + | - | + |
|  | *PeSEP4* | KF673860 | *Phalaenopsis equestris* | *SEP1*/*2*/*4*-like | + | + | + | + | + | - | + |
|  | *CgSEP1* | KF924272 | *Cymbidium goeringii* | *SEP3*-like | - | + | + | - | - | ND | ND |
|  | *CgSEP2* | KX347447 | *Cymbidium goeringii* | *SEP1*/*2*/*4*-like | + | - | - | - | - | ND | ND |
|  | *CgSEP3* | KX347448 | *Cymbidium goeringii* | *SEP3*-like | - | + | + | - | - | ND | ND |
|  | *CgSEP4* | KX347449 | *Cymbidium goeringii* | *SEP1*/*2*/*4*-like | + | + | - | + | + | ND | ND |
|  | *EpMADS3* | KJ002728 | *Erycina pusilla* | *AGL6*-like | + | + | - | + | ND | ND | ND |
|  | *EpMADS4* | KJ002729 | *Erycina pusilla* | *AGL6*-like | + | + | - | - | - | ND | ND |
|  | *EpMADS5* | KJ002730 | *Erycina pusilla* | *AGL6*-like | - | - | + | + | + | ND | ND |

^§^Modified from Aceto and Gaudio 2011; Mondragón-Palomino 2013; Teixeira da Silva et al. 2014.

‘‘-’’: Transcripts of gene were not detected

‘‘+’’: Transcripts of gene were detected

S; Sepal, P; Petal; L; Lip, C; Column, P/O; Pollinia/Ovary, VT; Vegetative tissue

ND: Detection of transcripts has not been performed

a: Root

b: Leaf

c: Shoot

**©^:^** Orchidstra Accession Number (Chao et al. 2017)
